# Hedonic hunger and eating behavior after low-carbohydrate versus low-fat diets in females with lipedema and obesity

**DOI:** 10.3389/fnut.2025.1716592

**Published:** 2025-12-17

**Authors:** Julianne Lundanes, Thea Gustad Naustvoll, Randi J. Tangvik, Catia Martins, Siren Nymo

**Affiliations:** 1 Department of Clinical and Molecular Medicine, Faculty of Medicine and Health Sciences, Norwegian University of Science and Technology (NTNU), Trondheim, Norway; 2 Nord-Trøndelag Hospital Trust, Clinic of Surgery, Namsos Hospital, Namsos, Norway; 3 Department of Clinical Medicine, Centre for Nutrition, University of Bergen, Bergen, Norway; 4 Department of Clinical Science, University of Bergen, Bergen, Norway; 5 Department of Nutrition Sciences, University of Alabama at Birmingham (UAB), Birmingham, AL, United States; 6 Center for Obesity Research, St. Olav's Hospital, Trondheim University Hospital, Trondheim, Norway; 7 Department of Public Health and Nursing, Faculty of Medicine and Health Sciences, Norwegian University of Science and Technology (NTNU), Trondheim, Norway

**Keywords:** restrained eating, food available, emotional eating, external eating, weight loss

## Abstract

**Introduction:**

Lipedema is a chronic female disease, characterized by an excessive accumulation of subcutaneous adipose tissue in the limbs and is commonly mistaken for obesity, although the two conditions often coexist. Obesity is associated with increased hedonic hunger and dysfunctional eating behavior. However, these aspects have not been investigated in females with lipedema and obesity.

**Objectives:**

The objective of this secondary analysis from a randomized controlled trial was to compare changes in hedonic hunger and eating behavior following two different low-energy diets, low-carbohydrate (CHO) or low-fat, in females with lipedema and obesity.

**Methods:**

Females with lipedema and obesity (body mass index (BMI) 30–45 kg/m^2^) were randomized to two different low-energy diets (1,200 kcal), low-CHO diet (LCD) (75 g CHO) or low-fat diet (180 g CHO) for 8 weeks. Hedonic hunger was assessed using the power of food scale (PFS) and eating behavior was assessed using the Dutch Eating Behavior Questionnaire (DEBQ) pre- and post-intervention.

**Results:**

A total of 70 females were included with a mean age of 47 years, and a BMI of 37 kg/m^2^. The LCD group reported a reduction in Food Present (*p* < 0.001) and in Aggregated Score (*p* = 0.035) from the PFS, while no changes were seen in the low-fat diet group, with changes in Food Present over time being significantly different between groups (*p* = 0.050). The low-fat diet group reported increases in Restrained Eating from the DEBQ (*p* = 0.036) while only the LCD group reported decreases in Diffuse Emotions (*p* = 0.040), however, no differences between groups were found.

**Conclusion:**

A LCD may induce more favorable changes in hedonic hunger and eating behavior than an isocaloric low-fat diet in females with lipedema, which may be related to altered metabolic signaling pathways related to satiety and reward.

## 1 Introduction

Lipedema is a chronic female disease characterized by painful adipose tissue accumulation in the lower limbs (1). Lipedema seems to have a genetic component (2), and has its onset during periods of hormonal fluctuations, such puberty, pregnancy and menopause (3). This suggests genetic and estrogen related etiology. The pathophysiology of lipedema is still not fully understood, however, it is thought to be multifactorial, and include adipocyte hypertrophy, inflammation, and impaired angiogenesis (3). Inflammatory dysregulation in lipedema may interfere with neuroendocrine appetite control mechanisms by modulating hypothalamic signaling pathways (4).

Appetite regulation is complex and involves a balance between internal biological cues that maintain energy balance (homeostasis) and external reward-driven factors (hedonic) (5). Hedonic appetite, driven by pleasure rather than physiological need (6), plays a significant role in modulating eating behavior (7), often leading to increased consumption of palatable foods independently of energy requirements (8). Dysfunctional eating behavior, such as disinhibition, emotional eating, and binge eating, is associated with increased energy intake and higher body mass index (BMI), and may contribute to obesity (9).

Females with lipedema have been overlooked and subjected to health-related stigma (10). The physical manifestations of lipedema can be distressing, contributing to a negative body image and unhealthy self-perceptions (11). Females with lipedema report lower quality of life than the general population (12, 13). A high prevalence of eating disorders (16%) has also been reported in females with lipedema (14), higher than the Norwegian general population (2%), however similar to people living with obesity (12%) (15). Differences in the cut-off scores and questionnaires used to assess eating disorders may prevent direct comparisons between population groups.

Low-carbohydrate diets (LCD) have emerged as a potential therapeutic tool in this populations (16). We (17, 18), as well as others (19, 20), have previously shown that a LCD is better in reducing pain in women with lipedema compared with an isocaloric low-energy low-fat diet. We have also shown that females with lipedema and obesity experience an increase in homeostatic hunger following weight loss with a low-fat diet, but not with a LCD (21), in line with findings in individuals with obesity without lipedema (22–25). However, how hedonic hunger and eating behavior change with different dietary interventions has not been explored in this patient group.

Ghrelin, an orexigenic hormone produced by the stomach, Skibicka and Dickson (26–29), and Abizaid (29) as well as insulin (30, 31) may regulate the mesolimbic reward system by modulating dopaminergic signaling. Given that an LCD can suppress ghrelin (21–23, 32, 33) and insulin (22, 25, 34, 35) secretion to a greater extent than a low-fat diet, as well as result in greater improvements in insulin sensitivity (36), a LCD could potentially affect food reward and appetite behavior differently than a low-fat diet. Additionally, females with lipedema seem to struggle more with emotional regulation compared with healthy controls (37).

Therefore, the main objective of this secondary analysis was to compare changes in hedonic hunger and eating behavior following two different low-energy diets, either an LCD or low-fat diet, in females with lipedema and obesity. We hypothesized that an LCD would reduce hedonic hunger and improve eating behavior compared to a low-fat diet in females with lipedema and obesity.

## 2 Methods

### 2.1 Study design

This paper represents a secondary analysis of a randomized clinical trial (RCT) comparing the effects of a low-energy LCD versus an isocaloric low-fat diet for 8 weeks on pain in females with lipedema. The main outcomes have previously been published (17). Approval was granted by the Regional Ethical Committee (REK 93888), and the study is registered in Clinicaltrials.gov (NCT04632810). All participants provided written, informed consent in accordance with the Helsinki Declaration prior to enrollment. Participants were randomized in a 1:1 ratio using block randomization with stratification based on BMI categories (30.0–34.9, 35.0–39.9, 40.0–44.9 kg/m^2^). Randomization was performed by a web-based randomization system developed and administered by the Faculty of Medicine and Health Sciences, Norwegian University of Science and Technology (NTNU), Trondheim, Norway. The data collection was performed using eFORSK, a web-based system developed and administered by Helse Midt-Norge IT (Central Norway Regional Health Authority’s IT department).

### 2.2 Study population

Females aged 18 to 75 years, with a BMI between 30 and 45 kg/m^2^ and diagnosed with lipedema were invited to participate in this study. They were diagnosed with lipedema by general practitioners or physiotherapists before inclusion, and the type and stage of lipedema were evaluated at baseline (BL) (38, 39). The inclusion criteria required weight stability for the past 3 months (±3 kg). The exclusion criteria included both acute and chronic kidney disease or failure, malignant or infectious disease, previous bariatric surgery, diabetes, psychiatric diseases, pregnancy, breastfeeding, use of medications known to affect body weight, insufficient proficiency in a Scandinavian language, and participation in other obesity or lipedema treatment program (except regular physiotherapy). No restrictions were placed on the use of compression garments/pulsators; thus, the participants who used these prior to the study were allowed to continue their use throughout the study period.

### 2.3 Dietary intervention

The participants were followed weekly by a clinical dietitian. The diets were matched for energy and protein but differed in carbohydrates (CHO) and fat composition. Both diets consisted of 1,200 kcal/day and 60 g of protein per day. The LCD consisted of 75 g (25 energy percentage (E%)) of CHO and 73 g (55 E%) from fats. The low-fat diet consisted of 180 g (60 E%) from CHO and 27 g (20 E%) from fat. For more information on the dietary intervention, see Lundanes et al. (17).

### 2.4 Outcome variables

The following explorative variables were assessed at BL and at the end of the intervention [week 9 (w9)].

#### 2.4.1 Hedonic hunger

Hedonic hunger was assessed using the power of food scale (PFS). The PFS has three different categories: Food Available, Food Present and Food Tasted, and an Aggregated Score as a summary of the three categories. The items are scored on a Likert scale from 1 = “I do not agree at all” to 5 = “I strongly agree” (40). Food Available represents food proximity for food readily available in the environment but not physically present. Food Present represents food proximity for food present but not tasted. Food Tasted represents food proximity for food when first tasted but not consumed (6, 40, 41). The PFS showed strong internal consistency, with Cronbach’s alpha values ranging from 0.81 to 0.91 (41).

#### 2.4.2 Eating behavior

Eating behavior was assessed using the Dutch Eating Behavior Questionnaire (DEBQ). The DEBQ consists of 33 items, and assesses Emotional, External, and Restrained Eating. The Emotional Eating category can be divided into two categories: Diffuse Emotions and Clearly Labelled Emotions. The items are scored on a Likert scale from 1 to 5, and ranges from “never” to “very often” (42). Emotional Eating represents the tendency to eat in response to negative feelings. External Eating represents how much a person eats as a response to an external cue, as seeing, smelling and the availability of food, regardless of physiological hunger. Restrictive Eating represents cognitive control over dietary intake, that is how much a person tries to limit or control how much he/she eats aiming to lose weight or remain weight stable. The subcategory of “Emotional Eating, Diffuse Emotions” represents eating behavior as a response to vague or indecisive feelings, such as boredom or worry, while the subcategory “Clearly Labelled Emotions” represents eating as a response to specific or known feelings such as sadness, anger, loneliness and anxiety (42, 43). The DEBQ demonstrated high internal consistency in individuals with obesity, with Cronbach’s alpha values ranging from 0.79 to 0.96 (44).

### 2.5 Statistical analysis

Statistical analysis was performed using Stata (StataCorp. Release 19. College Station, TX, USA), and data presented as mean and standard deviation (SD) and/or median and 25 and 75 percentiles. Data were checked for normality with Shapiro Wilk test and visual inspection of histograms. Statistical significance was assumed at *p* < 0.05. Changes from BL to w9 within each group were analyzed using Wilcoxon signed rank test, and differences in change between groups were estimated using Mann–Whitney U-test. Figures were generated using GraphPad Prism (Version 10.0.2 for Windows, GraphPad Software, Boston, Massachusetts, USA).

## 3 Results

### 3.1 Participants

A total of 70 females with lipedema and obesity were included in this secondary analysis, with a mean age of 47 ± 11 years and a mean BMI of 37 ± 5 kg/m^2^. Changes in body weight and body composition have already been published (17), but briefly, both the LCD and low-fat diet group experienced a significant weight loss (−10.2 kg and −7.4 kg, respectively), which was significantly greater in the LCD group compared to the low-fat diet group (−2.8 kg, *p* < 0.001).

### 3.2 Hedonic hunger

**Figure 1:**
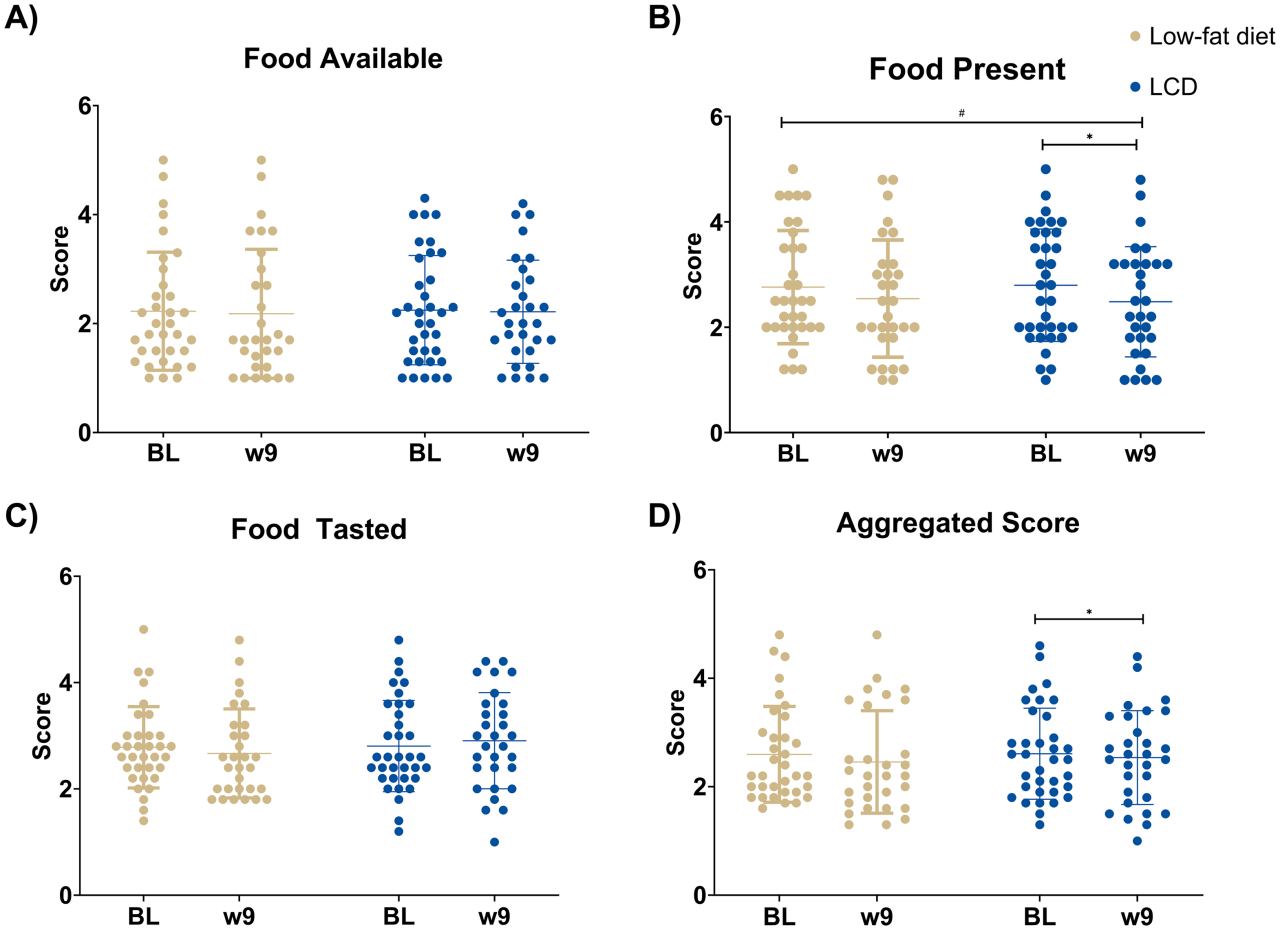
Hedonic appetite assessed using the power of food scale from the following subscales: **(A)** Food available, **(B)** Food present, **(C)** Food tasted, and **(D)** Aggregated score before and after two different low-energy diets: low-carbohydrate or low-fat diets. Data presented as individual values, mean and standard deviation. Changes over time within each group were analyzed using the Wilcoxon Signed Rank test, and differences in changes between groups were analyzed using the Mann–Whitney U-test. LCD: Low-carbohydrate diet. *p < 0.05, significant change from BL to week 9. ^#^*p* < 0.05, significant difference in change from baseline to week 9 between groups. BL, baseline; w, week.

Hedonic hunger from the PFS is presented in Figure 1 and Supplementary Table S1. The LCD group reported a reduction in Food Present (median values BL: 2.8 and w9: 2.4) (*p* < 0.001) and Aggregated Score (median values BL: 2.5 and w9: 2.5, *p* = 0.035), while no changes were found in the low-fat diet group. Changes in Food Present over time were significantly different between groups (*p* = 0.050).

### 3.3 Eating behavior

**Figure 2:**
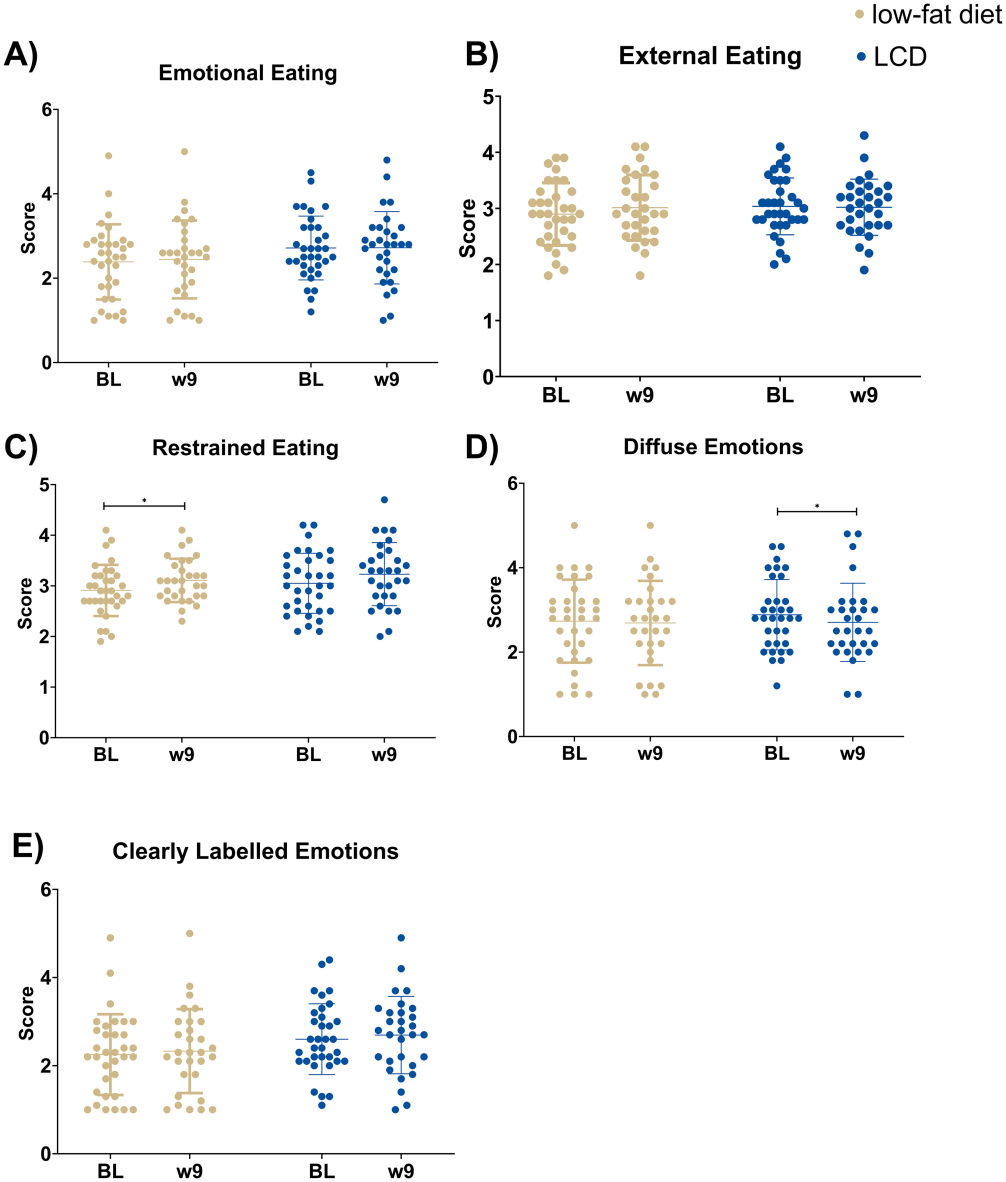
Eating behavior assessed using the Dutch Eating Behavior Questionnaire from the following subscales: **(A)** Emotional eating, **(B)** External eating, **(C)** Restrained eating, **(D)** Diffuse emotions, and **(E)** Clearly labelled emotions before and after two different low-energy diets: low-carbohydrate or low-fat diets. Data presented as individual values, mean and standard deviation. Changes over time within groups were analyzed using the Wilcoxon Signed Rank test, and differences in changes between groups were analyzed using the Mann–Whitney U-test. LCD, Low-carbohydrate diet.*p < 0.05, significant change from BL to week 9. ^#^*p* < 0.05, significant difference in change from baseline to week 9 between groups. BL: baseline. w, week.

Eating behavior scores from the DEBQ are presented in Figure 2 and Supplementary Table S2. Only the low-fat diet reported an increase in Restrained Eating (median values BL: 2.9 and w9: 3.1, *p* = 0.036), and only the LCD group reported a significant decrease in Diffuse Emotions (median values BL: 2.8 and w9: 2.5, *p* = 0.040). No differences between groups were found.

## 4 Discussion

The objective of this secondary analysis was to compare changes in hedonic hunger and eating behavior scores between two different low-energy diets: either a LCD or low-fat diet in females with lipedema and obesity. A reduction of Food Available and Aggregated Score from the PFS was seen in the LCD, and changes in Food Present scores were significantly different between groups. The low-fat diet group reported increases in restrained eating from the DEBQ, while the LCD group reported a decrease in Diffuse emotions. However, no differences between groups were found for any DEBQ categories.

The Food Present subscale from the PFS was significantly reduced following the LCD in this study, and this reduction was significantly larger compared to that seen in the low-fat diet group. The Aggregated Score was only reduced in the LCD group, and no differences between groups were found. Previous research has shown that diet-induced weight loss can reduce hedonic hunger (45–47). Aukan et al. (45) also found significant decreases in Food Present and Aggregated Score after diet-induced weight loss in a population with obesity.

The study by Aukan et al. (45) also found significant reductions in Food Available scores, which was not found in the present study. This discrepancy in findings may be due to differences in BL levels. In the present study, the BL scores of Food Available were lower (2.3) compared to Aukan et al. (~3.1) (45). Additionally, a study using pre-bariatric surgery data found higher levels of hedonic hunger from Aggregated Score (mean and SEM 3.1 ± 0.1) than those observed in the present study (mean and SD 2.5 ± 0.9) (48). Participant characteristics may also play a role, as this study included individuals with both lipedema and obesity, whereas the studies by Aukan et al. (45) and Nymo et al. (48) focused solely on participants with obesity. The participants from these studies also had a higher BMI (40 kg/m^2^ and 42 kg/m^2^, respectively versus 37 kg/m^2^ in the present study). Although obesity and lipedema share several features, they differ in pathophysiology. This may suggest that hedonic hunger is not a major concern in the lipedema population. Further studies are needed to examine hedonic appetite in females with lipedema.

The subcategory Food Present was reduced in the LCD group, however not the subcategory Food Available. This could possibly reflect neurocognitive adaptations to external food cues. The macronutrient composition of the diet is hypothesized to affect food cues by influencing satiety and cravings through a combination of learned associations and physiological signals (49).

CHO restriction in general and ketogenic diets in particular may modulate dopamine pathways, by affecting the endocrine circuity, namely leptin (50), ghrelin (26, 51) and insulin (52). We have previously reported, in the same participants that ghrelin concentrations are reduced postprandially after a LCD compared with low-fat diet (21). However, we did not measure leptin. Future studies should evaluate the potential role of insulin and insulin sensitivity on hedonics and eating behavior.

Darcey and colleagues investigated whether isocaloric reductions in dietary fat or CHO altered dopamine D2/3 receptor binding potential and neural activity in brain-reward regions in response to visual food cues in adults with obesity. Their findings indicate that fat restriction increases tonic dopamine in brain-reward regions and affects food choice in ways that may hinder dietary adherence (53). This is in line with our findings of a more favorable change in hedonic hunger in the LCD group.

A reduction in Diffuse Emotions was observed in the LCD group. The lack of change in the main category Emotional eating may be due to power restraints, or that a very specific type of emotional eating is present that is not captured by the main category. Diffuse Emotions is a subcategory of Emotional Eating, characterized by eating in response to worry, boredom, or idle (42). A decrease in this subcategory suggests improved emotional regulation and a reduced reliance on food as a coping mechanism (43). However, this finding is explorative, and the study may be underpowered for subscale-level analysis. Moreover, this finding needs to be replicated in future studies.

A ketogenic diet appears to promote emotional stability (54), potentially by increasing the inhibitory neurotransmitter GABA and reducing neuroinflammation. Evidence shows that ketogenic diets may increase GABA synthesis and reduces its breakdown, leading to a dampening of neuronal excitability that can stabilize mood (55). Simultaneously, ketogenic diets may mitigate neuroinflammation by reducing pro-inflammatory cytokines, modulating microglial activity, and influencing signaling pathways like NF-κB (56).

Emotional regulation has previously been investigated in females with lipedema (37). Al-Whardat et al. found that females with lipedema exhibited greater difficulties with emotional regulation compared to healthy controls (37). The fact that Diffuse Emotions improved exclusively in the LCD group contributes to the growing body of evidence supporting LCD as a potential treatment strategy for females with lipedema (17, 19, 21, 57–59). However, we did not find any differences between groups, and further research is needed to draw conclusions.

An increase in restrained eating was observed in the low-fat diet group, whereas no significant change was detected in the LCD group. This finding was somewhat unexpected, as both groups were subjected to energy restriction and would therefore be expected to report increased restrained eating behavior. However, diet-induced weight loss is commonly associated with increased homeostatic hunger (22, 23, 32), which in turn may reduce dietary adherence. Notably, nutritional induced ketosis, as a result of very low energy diets or LCDs, has been shown to attenuate the rise in hunger typically observed during weight loss (22–25). This pattern was also reflected in the current sample, where participants in the low-fat diet group reported increased hunger, while those in the LCD group reported increased feelings of fullness (21). It is therefore plausible that participants in the LCD group found it easier to adhere to the diet without perceiving their intake as overly restricted, while those in the low-fat group may have had to exert more conscious control over their eating behavior to achieve the same energy deficit due to increases in homeostatic hunger.

Hedonic hunger and eating behavior do not appear to be major concerns among females with lipedema and obesity, given the relatively normal BL levels observed. Obesity is defined as a BMI > 30 kg/m^2^; however, in the context of lipedema, BMI may be misleading due to the characteristic disproportion between the upper and lower body, as well as the metabolic risks associated with abdominal obesity (60). This may help explain the lower BL levels compared to other studies involving individuals with obesity without lipedema (45, 48). Furthermore, eating disorders were an exclusion criterion in this trial, meaning participants with significant dysfunctional eating behaviors were not included. It is important to note that comparing females with lipedema to individuals with obesity without lipedema was not an objective of this study. Future research should therefore explore this comparison more thoroughly. Additionally, upcoming studies are encouraged to use waist-to-height ratio instead of BMI to assess obesity, as this measure has been found to be more accurate in females with lipedema (60).

Body dissatisfaction seems to be a significant concern in females with lipedema (61), possible exacerbated by misdiagnosis and ineffective treatment strategies (62). Lipedema tissue has previously been described as resistant to energy restriction (63). This is a population group that has repeatedly been told by their general practitioners to lose weight (64), with no clear advice regarding which dietary strategy would be more adequate. Previous studies have shown that there is a clear lack of knowledge about lipedema among health care personnel (65). This might have contributed to the numerous unsuccessful weight loss attempts reported in this population, as well as experiences of self-blame and fat-shaming (64). Further studies should be performed to investigate body image in females with lipedema, and whether this improves following a LCD.

This study required considerable commitment from participants, both in terms of changes in their diet, as well as time. Such demands may have influenced the type of participants willing to enroll, potentially resulting in a sample that is healthier and less burdened by symptoms than the broader population with lipedema. Consequently, the findings may reflect characteristics of a more motivated and health-conscious subgroup, rather than being fully representative of all females living with lipedema. This selection bias should be considered when interpreting the results and underscores the need for future studies to include more diverse samples to capture the full spectrum of disease burden.

An LCD seems to be beneficial for females with lipedema (16), as shown by us (17, 18, 21, 58, 66), and others (19, 57, 59). An LCD seems to be beneficial in reducing pain (17–19), improve body composition (19, 58, 59), as well as prevent the increases in hunger otherwise seen with weight loss (21). The improvements seen in hedonic hunger add to the evidence suggesting a LCD as a potential treatment option for females with lipedema. The observed greater benefit of LCDs over low-fat diets in lipedema has direct translational relevance. Tailored nutritional counseling that incorporates principles of CHO restriction and ketosis may enhance symptom relief and patient adherence. Future biomarker-driven studies are warranted to clarify the mechanisms underlying the therapeutic effects of ketogenic diets and to identify metabolic and/or inflammatory signatures predictive of individual responses to such interventions.

This study has both strengths and limitations. This is a secondary analysis of a RCT, and to our knowledge the first study investigating hedonic appetite and eating behavior in lipedema patients. Both the PFS and DEBQ are validated tools and compliance with the diets was good. Limitations include the short intervention period. Additionally, despite the diets being designed to induce a similar weight loss; a larger weight loss was seen in the LCD group. The study was powered to investigate pain; hence it might be underpowered to look at changes in hedonic hunger and eating behavior over time, as well as differences between groups. Moreover, a Norwegian translation of the PFS and DEBQ have not been validated specifically in individuals with lipedema, and this may influence psychometric comparability with other populations. Finally, these analyses are explorative in nature, and interpretations need to be done with caution.

## 5 Conclusion

An LCD seems to be associated with more beneficial changes in hedonic hunger and eating behavior, compared with an isocaloric low-fat diet in females with lipedema. This adds to previous evidence on the superiority of LCD for body weight management in this population. LCDs may modulate hedonic appetite regulation and emotional eating behavior through altered metabolic signaling pathways related to satiety and reward. Future studies should investigate the long-term effects of LCDs in this patient population, as well as the underlying mechanisms including neuroendocrine assessment.

## Data Availability

The raw data supporting the conclusions of this article will be made available by the authors, without undue reservation.
