# Supplementary material for: Hedonic hunger and eating behavior after low-carbohydrate versus low-fat diets in females with lipedema and obesity
Source: Front Nutr. 2025 Dec 17;12:1716592. doi: 10.3389/fnut.2025.1716592 (PMC12753418; doi:10.3389/fnut.2025.1716592)
Supplement: Supplementary file 1 [file Table_1.docx]

| Supplementary Table 1. Power of food scale scores before and after low-energy, low-carbohydrate versus low-fat diets | | | | | | | | | | | | | |
| --- | --- | --- | --- | --- | --- | --- | --- | --- | --- | --- | --- | --- | --- |
|  |  | **Baseline** | | | | | **Week 9** | | | | | P value within groups^1^ | P value between groups^2^ |
|  | Grp | Percentiles | | | mean | SD | Percentiles | | | mean | SD |  |  |
|  |  | 25 | 50 | 75 |  |  | 25 | 50 | 75 |  |  |  |  |
| Food Available | LCD | 1.3 | 2.2 | 3.2 | 2.3 | 1.0 | 1.5 | 2.0 | 2.8 | 2.2 | 0.9 | 0.125 | 0.563 |
|  | Low-fat | 1.5 | 1.9 | 2.7 | 2.2 | 1.1 | 1.2 | 1.7 | 3.0 | 2.2 | 1.2 | 0.567 |  |
| Food Present | LCD | 2.0 | 2.8 | 3.8 | 2.8 | 1.1 | 1.8 | 2.4 | 3.3 | 2.5 | 1.0 | **<0.001** | **0.050** |
|  | Low-fat | 2.0 | 2.5 | 3.5 | 2.8 | 1.1 | 1.8 | 2.4 | 2.4 | 2.5 | 1.1 | 0.167 |  |
| Food tasted | LCD | 2.2 | 2.6 | 3.6 | 2.8 | 0.9 | 2.4 | 2.9 | 3.6 | 2.9 | 0.9 | 0.310 | 0.257 |
|  | Low-fat | 2.4 | 2.7 | 3.0 | 2.8 | 0.8 | 2.0 | 2.5 | 3.2 | 2.7 | 0.8 | 0.582 |  |
| Aggregated score | LCD | 1.9 | 2.5 | 3.3 | 2.6 | 0.8 | 1.9 | 2.5 | 3.3 | 2.5 | 0.9 | **0.035** | 0.854 |
|  | Low-fat | 1.9 | 2.2 | 3.0 | 2.6 | 0.9 | 1.7 | 2.2 | 3.5 | 2.5 | 0.9 | 0.151 |  |
| Differences in change between groups were analyzed using Mann-Whitney U-test and Wilcoxon signed-rank test. LCD: Low-carbohydrate diet. SD: Standard deviation.^1^ P value for changes from baseline to week 9 within each group. ^2^P value for differences between groups in change over time. | | | | | | | | | | | | | |
