# Supplementary material for: Hedonic hunger and eating behavior after low-carbohydrate versus low-fat diets in females with lipedema and obesity
Source: Front Nutr. 2025 Dec 17;12:1716592. doi: 10.3389/fnut.2025.1716592 (PMC12753418; doi:10.3389/fnut.2025.1716592)
Supplement: Supplementary file 2 [file Table_2.docx]

| Supplementary Table 2. Dutch eating behavior questionnaire before and after low-carbohydrate and low-fat diets. | | | | | | | | | | | | | |
| --- | --- | --- | --- | --- | --- | --- | --- | --- | --- | --- | --- | --- | --- |
|  |  | **Baseline** | | | | | **Week 9** | | | | | **P value within group^1^** | **P value between groups^2^** |
|  | Grp | Percentiles | | | mean | SD | Percentiles | | | mean | SD |  |  |
|  |  | 25 | 50 | 75 |  |  | 25 | 50 | 75 |  |  |  |  |
| Emotional eating | LCD | 2.3 | 2.6 | 3.2 | 2.7 | 0.8 | 2.2 | 2.8 | 3.2 | 2.7 | 0.9 | 0.432 | 0.713 |
|  | Low-fat | 1.8 | 2.5 | 2.8 | 2.4 | 0.9 | 1.8 | 2.6 | 2.9 | 2.4 | 0.9 | 0.962 |  |
| Restrained eating | LCD | 2.5 | 3.0 | 3.5 | 3.1 | 0.6 | 2.8 | 3.3 | 2.6 | 3.2 | 0.6 | 0.262 | 0.413 |
|  | Low-fat | 2.7 | 2.9 | 3.2 | 2.9 | 0.5 | 2.8 | 3.1 | 3.4 | 3.1 | 0.4 | **0.036** |  |
| External eating | LCD | 2.8 | 3.0 | 3.5 | 3.0 | 0.5 | 2.7 | 3.1 | 3.3 | 3.0 | 0.5 | 1.000 | 0.129 |
|  | Low-fat | 2.5 | 2.9 | 3.3 | 2.9 | 0.6 | 2.6 | 2.9 | 3.5 | 3.1 | 0.6 | 0.061 |  |
| Diffuse emotions | LCD | 2.3 | 2.8 | 3.3 | 2.9 | 0.8 | 2.3 | 2.5 | 3.0 | 2.7 | 0.9 | **0.040** | 0.314 |
|  | Low-fat | 2.0 | 2.8 | 3.3 | 2.7 | 1.0 | 2.3 | 2.8 | 3.3 | 2.7 | 1.0 | 0.364 |  |
| Clearly labelled emotions | LCD | 2.1 | 2.5 | 3.1 | 2.6 | 0.8 | 2.1 | 2.7 | 3.2 | 2.7 | 0.9 | 0.945 | 0.785 |
|  | Low-fat | 1.4 | 2.3 | 2.8 | 2.3 | 0.9 | 1.8 | 2.3 | 3.0 | 2.3 | 0.9 | 0.736 |  |
| Differences within group were analyzed using Wilcoxon Signed Rank test, and differences in change between groups were analyzed using Mann-Whitney U-test. LCD: Low-carbohydrate diet. SD: Standard deviation. ^1^P values for changes from baseline to week 9 within each group (Wilcoxon Signed Rank test). ^2^P values for differences between groups in changes over time (Mann Whitney U-test). | | | | | | | | | | | | | |
